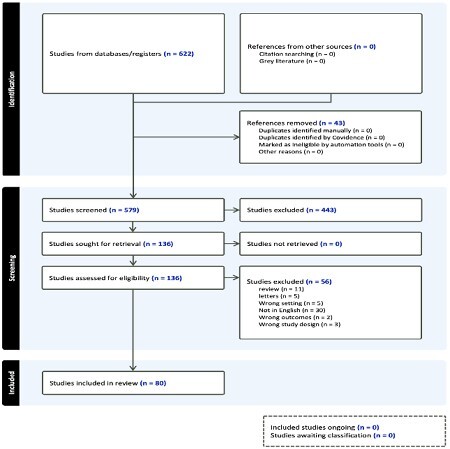# 801 Post-Burn Breast Reconstruction: A Systematic Review of Techniques

**DOI:** 10.1093/jbcr/irae036.341

**Published:** 2024-04-17

**Authors:** Riley Marlar, Tomer Lagziel, Ying Ku, Sarah N Bishop

**Affiliations:** Cleveland Clinic Foundation, Cleveland, Ohio; Department of Plastic Surgery, Johns Hopkins School of Medicine, Baltimore, MD; Cleveland Clinic, Cleveland, OH; Cleveland Clinic Foundation, Cleveland, Ohio; Department of Plastic Surgery, Johns Hopkins School of Medicine, Baltimore, MD; Cleveland Clinic, Cleveland, OH; Cleveland Clinic Foundation, Cleveland, Ohio; Department of Plastic Surgery, Johns Hopkins School of Medicine, Baltimore, MD; Cleveland Clinic, Cleveland, OH; Cleveland Clinic Foundation, Cleveland, Ohio; Department of Plastic Surgery, Johns Hopkins School of Medicine, Baltimore, MD; Cleveland Clinic, Cleveland, OH

## Abstract

**Introduction:**

Breast burns, whether thermal, chemical, or electrical, represent a significant subset of burn injuries. The psychological and physical implications of these burns can be profound, particularly given the breast's anatomical and socio-cultural significance. Post-burn breast reconstruction is not only crucial for functional recovery but also for emotional and psychological rehabilitation. With evolving techniques over the years, there is a pressing need to systematically assess and consolidate information to guide future therapeutic directions.

**Methods:**

A systematic search of PubMed, EMBASE, and the Cochrane Library was conducted following PRISMA guidelines, including studies published up to September 2022. Inclusion criteria comprised articles focusing on surgical techniques, outcomes, and complications of breast reconstruction following burn injuries. Data extraction included study characteristics, patient demographics, burn details, reconstructive techniques, outcomes, and complications. A qualitative synthesis was performed due to the heterogeneous nature of the included studies.

**Results:**

The search yielded 80 relevant studies, emphasizing a myriad of reconstructive options including autologous tissue transfer, acellular dermal matrices, tissue expanders, and implant-based reconstructions. Flap-based reconstructions were exemplified for optimal aesthetic and functional outcomes with reduced donor site morbidity. However, each technique's success is contingent upon the burn’s extent, location, and patient-specific factors. Complications were varied, with infection, flap failure, and contracture being prevalent. Notably, advancements in regenerative medicine and bioengineering, such as tissue engineering and fat grafting, have shown promising results in restoring breast contour and improving scar quality.

**Conclusions:**

This systematic review sheds light on the multifaceted domain of post-burn breast reconstruction. The key takeaway is the importance of individualized treatment plans, factoring in patient needs, burn characteristics, and available resources. The advent of regenerative techniques is certainly promising, emphasizing the need for continuous research to refine and innovate reconstruction methodologies further.

**Applicability of Research to Practice:**

The evidence underscores the versatility of available reconstructive techniques, aiding clinicians in tailoring interventions based on individual patient needs, burn extent, and aesthetic considerations. This insight enables practitioners to refine surgical strategies, optimize outcomes, and mitigate complications. The highlighted advancements in regenerative medicine provide a framework for integrating innovative solutions in clinical practice, enhancing the scope of holistic burn management and improving patient quality of life.